# Body composition as a marker of performance and health in military personnel

**DOI:** 10.3389/fspor.2023.1223254

**Published:** 2023-12-18

**Authors:** Lynn Cialdella-Kam, Taylor K. Bloedon, Michael S. Stone

**Affiliations:** ^1^ Warfighter Performance Department, Naval Health Research Center, San Diego, CA, United States; ^2^ School of Applied Health, Cal Poly Humboldt, Arcata, CA, United States; ^3^ Military and Veterans Health Solutions, Leidos, San Diego, CA, United States

**Keywords:** body fat, disordered eating, lean body mass, operational readiness, warfighter performance, weight cycling

## Abstract

**Introduction:**

Body composition standards are set to ensure operational readiness in active-duty military personnel. To meet body composition standards, some individuals, however, may engage in unhealthy weight control behaviors (i.e., weight cycling and disordered eating). The objectives of this review are to: (1) evaluate the evidence regarding body composition and the associations to physical and military specific performance; (2) discuss body composition and potential health consequences; and (3) examine the evidence of weight cycling and disordered eating behaviors in military personnel for weight control.

**Methods:**

A systematic search to identify peer-reviewed research articles was conducted in PubMed on 2/20/2023 using Medical Subject Headings (MeSH) including but not limited to “Military Personnel”, “Tactical Athlete”, “Weight Loss”, “Body Composition”, and “Weight Cycling”.

**Results:**

A total of 225 research articles were identified. The list was narrowed down to articles from the last 20 years (2003–2023) in military personnel. Only studies in which percent body fat was directly measured were included resulting in 17 research articles for this review.

**Discussion:**

Evidence-based research is limited on the relationship between body composition and operational readiness. Weight cycling and disordered eating behaviors also has been reported for weight control, yet additional research is needed. Specifically, future research should focus on female service members, racial and ethnic differences, age, and postpartum status and include other service branches (i.e., Air Force and Navy). A comprehensive survey on weight cycling, disordered eating, and weight management would be valuable to determine the prevalence and extent of this issue. This information along with performance data would guide policy makers on the relevance and appropriateness of existing body composition standards.

## 1. Introduction

Body composition is often used as a marker of performance and health in active-duty military personnel and in athletes. The manipulation of body composition for athletics is a common practice to decrease the energy cost of doing work in gravitational sports (e.g., running and cycling), improve speed and agility in team sports, maximize power to body weight ratio in explosive power sports, and increase leanness in aesthetic sports (1). In the military, body composition standards are set to ensure operational readiness and decrease the risk of injuries, illnesses, and chronic disease (2). The overweight and obesity epidemic, however, is a challenge for the military (3). In 2004, a committee of experts compiled a report on weight management in face of this epidemic citing issues such as retention and recruitment of military personnel (4). The prevalence of obesity in United States (U.S.) adults (≥20 years) was estimated to be 32.2% in 2004 (5). This problem has since grown with recent estimates that ∼41.9% of U.S. adults (≥20 years) have obesity (6). The challenges for the military include a smaller recruiting pool [i.e., ∼19% of young adults (20–24 years) do not qualify for service due to having obesity (7)], increased risk of injuires and compromised physical readiness, and potential greater heatlh cost burden (3). The Department of Defense (DoD) recently updated instructions (DoD Instructions 1308.03) on physical performance and body composition as service branches such as the Marines re-evaluate body composition standards (8).

Currently, each U.S. service branch determines the specifics of their body composition requirements, and body composition is assessed either annually or bi-annually. With >1.1 million active-duty members in service, U.S. military branches are interested in quick and reliable methods. Weight-to-height screens and circumference measurements have been the preferred methods to determine if a service member meets body composition standards. Estimates of percent body fat (%BF) are calculated from military specific equations using circumference measurements, which have been validated against %BF measures using dual x-ray absorptiometry [DXA; e.g., considered the “gold standard” for body composition (9)]. A maximum %BF has been set based on sex, age, and service branch to ensure operational readiness and health of our forces. While the appropriateness and validity of body composition assessment methodology are beyond the scope of this article, the use of %BF and body composition as an indicator of performance and health requires further evaluation.

The practice of using weight and body composition as a measure of physical readiness and health is widely accepted in the medical and sports fields, although not without risk. Weight cycling (the repetitive loss and gain of body weight) and disordered eating behaviors employed to manage weight and body composition, has been well documented in athletes, particularly those in weight sensitive sports (10, 11). Less data is available regarding military personnel and the associated health consequences. Thus, the objectives of this review are to: (1) evaluate the evidence regarding body composition and associations to physical and military specific performance; (2) discuss body composition and potential health consequences; and (3) examine the evidence of weight cycling and disordered eating behaviors in military personnel for weight control.

## 2. Methods

A systematic search to identify peer-reviewed research articles was conducted in PubMed on 2/20/2023 using Medical Subject Headings (MeSH) when available with exceptions noted. Search criteria included “Firefighters” OR “Emergency Personnel” OR “Military Personnel” OR “Tactical Athlete” AND “Weight Loss” OR “Body Composition” OR “Feeding and Eating Disorders” OR “Rapid Weight Loss”. Of these terms, all were MeSH except for “Tactical Athlete” and “Rapid Weight Loss”. A separate search on “Weight Cycling”, a MeSH term introduced in 2022, was also conducted. A total of 225 research articles were identified. The list was narrowed down to articles from the last 20 years (2003–2023) in military personnel. Research studies on special military populations [i.e., Reserve Officers’ Training Corps (ROTC), reserves, national guards, conscripts, and Veterans] were excluded because these populations are not considered active duty (i.e., full time or have completed basic training). In addition, interventions studies (e.g., nutrition and exercise interventions targeted at weight loss and/or health and performance improvements) and disease specific research (e.g., metabolic disease, cancer, diabetes) were excluded. Finally, only research studies in which %BF was directly measured were included. Based on the listed inclusion criteria, 17 research articles were included in this review.

## 3. Body composition and performance in military personnel

The relationship between body composition and physical performance has been examined in nine studies in the active-duty military personnel, five of which are in soldiers (12–16), two in Marines (17, 18), one in sailors (19), and one in general military personnel (20). The discussion of research studies is organized by body composition methodology. The reliability and validity of these methods are beyond the scope of this review, and the reader is referred to (21, 22) for more information.

### 3.1. Air displacement plethysmography studies

Air displacement plethysmography (i.e., BodPod) was used to assess body composition in four studies, two in 101st Airborne U.S. Army soldiers (12, 13) and two in U.S. Marines (4, 19). Assessments of physical performance (i.e., aerobic and anaerobic capacity, muscular strength and power, and military specific tasks and measures) are summarized in Table 1.

**Table 1:** Body composition (BC) and physical performance measurements in military studies.

Studies	BC assessment method	Aerobic capacity (VO_2_max)/anaerobic capacity	Muscular strength and power assessments	Task or military specific measurements
Abt et al. (12)	Air displacement plethysmography	Incremental treadmill protocol and lactate threshold for aerobic capacity Wingate protocol for anaerobic capacity	Peak isokinetic torque (Biodex; concentric/concentric at 60°/second) of 5 repetitions at 100% maximal effort for knee extension/flexion, shoulder internal/external rotators, and torso rotators	None
Allison et al. (17)	Air displacement plethysmography	Incremental treadmill protocol and lactate threshold for aerobic capacity Wingate protocol for anaerobic capacity	Peak isokinetic torque (Biodex; concentric/concentric at 60°/second) of 5 repetitions at 100% maximal effort for knee extension/flexion, shoulder internal/external rotators, and torso rotators and isometric ankle eversion/inversion using hand-held dynamometer	None
Beck et al. (16)	Dual energy x-ray absorptiometry	None	A pack lift (median mass = 40.0 kg) to a 1.5 m platform and two box lifts to 1.5 m (median mass = 37.5 kg) and 1.3 m platform (median mass = 40.0 kg). Participants wore combat uniform including a weighted vest during each lift, which progressed from a 15 kg starting weight until failure (increments of 5 kg to max weight of 60 kg) (all lifts from the ground to between knuckle and chest height, pausing, taking one step forward and placing on platform)	Pack was standard military pack
Crawford et al. (13)	Air displacement plethysmography	Incremental treadmill protocol and lactate threshold for aerobic capacity Wingate protocol for anaerobic capacity	Peak isokinetic torque (Biodex; concentric/concentric at 60°/second) of 5 repetitions at 100% maximal effort for knee extension/flexion and shoulder internal/external rotators	Army Physical Fitness test (APFT), (maximum number of push-ups and sit-ups in a 2-minute time period and 2-mile timed run) conducted according to protocol
Orantes-Gonzales et al. (14)	Bioelectrical impedance	20 m shuttle run test for aerobic capacity	Squat jump test for leg power (a triaxial force platform sampling at 1,000 Hz [AMTI OPT464508HF, Watertown, USA] was used with Accu-Power 3.0 software [Advanced Mechanical Technology, Inc., Watertown, MA, USA] to measure the jump height)	Obstacle course containing six military specific obstacles (4.5 m cargo net climb, balance log, steel wire balance, 10 m tunnel crawl, 15 m rope climb
Pihlainen et al. (15)	Bioelectrical impedance	3,000 m running test for aerobic capacity Anaerobic capacity: none	Maximal isometric force of the lower and upper extremities extensor muscles measured bilaterally in a sitting position by using electromechanical dynamometer Standing long jump to assess explosive force production of the lower extremities. Sit-up, push-up and pull-up tests for muscle strength	Military simulation test consisting of four consecutive 6.2 m rushes, 11.3 m low crawl, 21.8 m sprint, 21.8 m run and jump over three 40 cm obstacles separated by 5 m, kettlebell (16 kg × 2) lift and carry (2.5 m × 4), 42.4 m zig zag run, 24 m mannequin (5 kg) drag
Ricciardi et al. (20)	Bioelectrical impedance	Incremental treadmill protocol and lactate threshold for aerobic capacity Anaerobic capacity: none	Physical performance battery: pull-ups, hang-time, and stair-stepping	All physical tasks were performed with and without body armor
Royer et al. (18)	Air displacement plethysmography	Incremental treadmill protocol for aerobic capacity Wingate protocol for anaerobic capacity	Peak isokinetic torque (Biodex; concentric/concentric at 60°/second) of 5 repetitions at 100% maximal effort for knee extension/flexion and trunk extension/flexion	None
Sporis et al. (19)	Skinfold measurements	Incremental treadmill protocol for aerobic capacity Sprint over distance of 5, 10, and 20 m for anaerobic performance	Countermovement jump (muscle power from force platform), squat jump (muscle power from force platform), standing long jump (distance)	None

Crawford et al. (2011) grouped male soldiers (*n* = 99) into two groups based on %BF: group 1 = %BF ≤18 (i.e., those who met DoD standards) and group 2 = %BF >18 (13). Of the physical performance metrics assessed, VO_2_max (ml/kg/min), anaerobic capacity, push-up scores and peak torque [% body weight (BW)] were found to be greater in group 1 compared to group 2. When peak torque was normalized to fat free mass (FFM; kg), only peak isokinetic shoulder internal rotation remained higher. No differences in performance were found for sit-ups and the 2-mile run. Thus, overall FFM may be a better indicator of performance capabilities than %BF.

Although the relationship between body composition and performance were not directly evaluated, Abt et al. (2016) assessed similar metrics and divided 253 U.S. male soldiers into groups categorized by age (20–24, 25–29, 30–34, 35–39, 40–44 years) and years of military service [1–5, 6–10, 11–15 years (12)]. Body composition was also evaluated among groups but was not directly compared to performance metrics. Reported differences included higher %BF in older compared to younger age groups (30–34 years vs. 20–24 and 25–29 years) and in soldiers with longer compared to shorter military tenure (11–15 years of service vs. 1–5 and 6–10 years). In measures of physical performance, VO_2_max (ml/kg/min) was higher in the youngest age group (20–24 years vs. 25–29; 35–39; 40–44 years). Among years of service, VO_2_max (ml/kg/min) and lactate threshold (i.e., the inflection point where blood lactate levels increase rapidly) were greater in those with 1–5 compared to those with 11–15 years of service. However, the researchers did not assess the relationship between %BF and performance metrics in this study. Thus, no conclusions can be drawn regarding the impact of %BF on performance.

In U.S. Marines, Royer et al. (18) compared physical performance in males in two Special Operation Forces (SOF) job categories: Critical Service Operators (CSOs, *n* = 164; highly trained in combat and tactics) and enablers [*n* = 51; enablers; specialists in areas such as medical care, intelligence, and communication (18)]. No differences were found between groups for absolute FFM (kg), but FFM index (i.e., normalized to height and calculated as FFM [kg]/height [m^2^]) was greater in CSOs vs. enablers. Anaerobic capacity, VO_2_max (ml/kg/min), and peak torque (%BW) for assessed muscle groups was found to be greater in CSOs vs. enablers. While some association was found between indices of body composition (e.g., FFM index) and physical performance, the direct relationship between %BF, job categorization, and performance metrics remains unclear.

Allison et al. (17) also conducted a study in U.S. Marines [male (*n* = 218), female (*n* = 84)] to examine the relationship between body composition and performance metrics (17). Using hierarchical cluster, Marines were grouped based on two Combat Fitness Test (CFT) events: Maneuver Under Fire (MUF) and Movement to Contact (MTC). Cluster 1 (C1; *n* = 82, 66 females, 16 males) had lower scores on all physical performance metrics (mean not reported by cluster) compared to Cluster 2 (C2; *n* = 212, 18 females, 194 males). Differences in %BF and performance measurements were found between males and females (males performing higher and having lower FM and greater FFM), which likely driven by physiological sex differences (23). Additionally, women in CF1 had significantly higher %BF and Fight Load Index (FLI; a ratio of FM to FFM relative to fight load indicative of military task performance) compared to those in C2; however, there were no other differences within groups or between performance metrics. Thus, females with higher %BF and FLI may perform more poorly during MUF and MTC exercises; however, evidence is lacking to conclude that %BF alone is associated with these scores.

### 3.2. Segmental multi-frequency bioelectrical impedance research

Bioelectrical impendence was used to assess body composition in three studies, two in soldiers (14, 15) and one in an unidentified service branch (20). A key aspect of these findings was the use of military stimulated tasks as a performance indicator.

In 81 male Finnish soldiers, Pihlainen et al. (15) assessed associations between physical fitness, body composition and time to complete the operationally relevant Multiple Stimulated Task course [MST, Table 1, typical Solider maneuvers and tasks with length = 242.5 m (15)]. A Dead Mass Ratio (DMR) was calculated by diving body mass (BM; kg) by the sum of fat mass (FM; kg) and load carry weight {kg [i.e., DMR = BM/(FM + load carry weight)]}. Researchers reported that DMR was the strongest predictor of MST, which was correlated with %BF and skeletal muscle mass (kg). Association between %BF and the other performance tests were not assessed. Similarly, Orantes-Gonzales (14) measured body composition and physical performance and associations to completion time of a military training obstacle course (with and without combat armor, Table 1) in male Spanish soldiers (*n* = 40) (14). The best predictors of course completion time with and without body armor were body mass index (BMI) of 25–30 kg/m^2^ and VO_2_max >55 ml/kg/min. Investigators reported no associations between %BF and course completion time.

In U.S. military personnel (*n* = 34, 17 females, 17 males, branch unspecified), Ricciardi et al. (2007) examined the effect of sex and %BF on completing a treadmill test with or with body armor. The single best predictor of test completion was %BF; sex, however, had no outcome effects (20). Taken together, %BF may be associated with military specific task performance. In the three studies discussed, each of the military task specific measures, however, differed, and only Ricciardi et al. (2007) included female warfighters. Further research is needed to validate findings to broader military populations.

### 3.3. Other body composition methods

In the only study to use DXA, Beck et al. (2019) evaluated the associations between military lifting task performance (Table 1) and anthropometrics in Australian soldiers [*n* = 63, 21 females, 42 males (16)]. Researchers assessed associations between lift performance (i.e., maximum weight lifted) and lean mass (LM) and FM for whole-body, as well as LM for trunk, lower-arm, upper-arm, and legs. All LM indices were strongly correlated to lift performance (r range 0.92 −0.99) while whole body FM was not. Additionally, when controlling for upper-arm and leg LM, no significant effects of stature or sex were found. Thus, increases in region specific LM may be beneficial for performance in military lifting tasks.

In Croatian sailors (*n* = 42 males), Sporis et al. (2011) estimated %BF via skinfold measurements [using Jackson-Pollock (24) equation] and examined the associations to fitness test performance [Table 1 (19)]. All performance measurements were negatively correlated to %BF (r range −0.42 to −0.67). While this was the only study in Navy personnel, key limitations in applicability to a broader military population are the use of skinfold measurements for %BF and a lack of female participants.

### 3.4. Summary

The link between body composition and military performance is not well established. Findings from a recent body composition standard study reported by Potter et al. (2022) in a technical report documented a downward trend between %BF (DXA) and performance measurements (physical fitness test and combat fitness test scores) in Marines (1,436 men and 737 women). This association, however, did not reach statistical significance (25). A positive aspect of this research is the inclusion of a large number of female Marines. Additional research, however, is needed in the female warfighter in general and in other service branches (i.e., Air Force and Navy). Based on the current research, total FFM appears to be a greater predictor of performance than %BF. Other factors related to body composition such as racial and ethnic differences, postpartum status, and age should be considered.

## 4. Body composition, health, and weight cycling in military personnel

The relationship between body composition, weight cycling, and health is a complex topic as health is often defined using a range of variables. In military personnel, body composition and health has only been examined in six studies (26–31) whereas weight cycling has only been examined in the context of disordered eating (32, 33). Thus, evidence in the military population is limited.

### 4.1. Body composition and health

For body composition and health, we assessed research on general health outcomes as well as musculoskeletal injury (MSKI) risk. For general health, two research studies on military personnel were identified, one in soldiers (26) and one in pilots (27). Using multi-frequency segmental BIA to determine body composition, Tingelstad et al. (2018) enlisted 331 soldiers (208 men and123 women) from Canadian Armed Forces and assessed the impact of body composition on pro-and anti-inflammatory markers, by age (19–29, *n* = 122; 30–39, *n* = 81; 40–49, *n* = 86; and 50–59 years, *n* = 42) and sex. Overall, women were found to have higher %BF (30.2 ± 8.0 vs. 21.7 ± 8.3), and the youngest age group (19–29 years) had lower %BF (20.6 ± 8.9) and higher lean body mass (63.7 ± 10.7) compared to other age groups (26). Higher levels of adiponectin and lower levels if IL-18 were detected in females compared to males. Levels of CRP, IL-18, IL-2 increased with increasing age, and CRP and IL-18 increased with higher adiposity. In military helicopter pilots (*n* = 22; 37.22 ± 7.90 years), Cárdenas et al. (2020) assessed total FM and visceral adipose tissue (VAT) via DXA, and the impacts on cognitive health via integrity of white matter (WM). All pilots were considered normal weight (BMI = 25.48 ± 2.49 kg/m^2^), and a higher total FM was linked to improved WM integrity while higher VAT was associated with decreased WM integrity (27). In previous reports, a higher BMI has been linked to poor cognitive health outcomes, although, as demonstrated in this study, the type and location of accumulated fat may be a better indicator than BMI alone.

The risk of injury may also be considered a health outcome. As summarized in a recent systematic review with meta-analysis (34), a higher BMI (i.e., > 25 kg/m^2^; classified as overweight or obese) is associated with musculoskeletal injuries (MSKI) in active duty military personnel; however, while an indicator of injury risk in 14 of the 34 studies assessed, BMI is not a direct measure of body composition (i.e., FM, FFM). Further, only two of the 14 studies assessed associations in personnel beyond the recruitment period (i.e., ≥ 90 days post accession) (35, 36). Bertrandt et al. (2020) examined lower body muscle symmetry in relation to MSKI in Polish Armed Forces soldiers (*n* = 504) and found those with asymmetrical distribution of lower body lean mass were at greater MSKI risk, as assessed by self report injuries and functional movement screen (FMS) (28). In a group of Explosive Ordnance Disposal (EOD) Technicians (*n* = 64), Hernandez and colleagues (2020) found that those with higher %BF, as assessed via DXA, and lower cardiorespiratory fitness (VO_2_ max) had lower FMS and Y-Balance Test scores and thus greater injury risk (29). Two other studies in SOF personnel [i.e., US Army SOF, Navy Sea, Air, and Land (SEALs), Air Force; *n* = 821 combined total] assessed potential associations between MSKI risk and different strength, balance, and physiological laboratory assessed characteristics (30, 31). Body composition was measured using air displacement plethysmography in both studies and %BF was not related to injury risk. While excessive %BF has been cited as an important factor for MSKI (37), research is still limited on associations between direct measures of %BF and these injuries in active duty, non-recruit military populations.

### 4.2. Weight cycling and health in military personnel

Weight cycling is a term used to describe a repeated pattern of weight change (e.g., weight loss to achieve a certain body weight followed by weight regain). A precise definition of weight cycling (e.g., how much weight loss/gain or how often) has not been developed. The consequences of weight cycling and health, to our knowledge, has not been examined in the military personnel. However, weight cycling in the general population has been associated with poorer musculoskeletal health, poor sleep quality, and increased risk for chronic diseases. Specifically, weight cycling has been linked to an increased risk of low muscle mass by 3.8-times, lower strength by 6.3-times, and sarcopenia by 5.2-times. During periods of weight loss, particularly rapid weight loss, LM also declines, and fat accumulation is more prominent when weight regain is rapid, a common pattern in weight cycling. Over time, this results in an overall increase in FM, a lower resting metabolic rate (RMR; kcal/day), and lower LM (38), potentially increasing risk for injuries and health complications (39). In addition, Zou et al. (2019) reported in a meta-analysis that weight fluctuation was associates with an increased risk of 49% for CVD morbidity, 35% for hypertension, 36% for CVD mortality, and 41% for all-cause mortality (40). The same group also reported that individuals who engaged in weight cycling had a 23% increased risk for developing type 2 diabetes (41). In women, weight cycling has been demonstrated to increase insulin resistance and cause unfavorable change in blood lipid profile with a greater impact on those at higher weights. Furthermore, the relationship between weight cycling on sleep was recently examined by Cao et al. (2021). After each weight cycling episode, women in this study reported poorer sleep quality [i.e., shorter sleep duration, longer sleep onset latency, greater insomnia severity, more sleep disturbances, lower sleep efficiency, and higher sleep medication use (42)]. Thus, long term weight cycling may increase health risks and counter performance and fitness goals.

Another concern with weight cycling is the increased risk of developing a clinical eating disorder. Disordered eating refers to behaviors such as fasting, dieting, and diuretic/laxative use (43), and engagement in these behaviors can lead to development of clinical eating disorder [i.e., defined by Diagnostic Statistical Manual of Mental Disorders 5th Edition (DSM-5) (44, 45)]. In the military population, research in which the association among weight cycling, disordered eating, and body composition was assessed is very limited (32, 33). Flatt et al. (2021) analyzed responses to an online eating disorder screening instrument, the Stanford-Washington Eating Disorders screen (SWED, 18 items) collected from 1,744 military personnel and veterans (73% female, 25% male, 2% non-binary) and 111,644 civilians (91% female, 6% male, 3% non-binary). Participants were asked in the last three months if they had engaged in binge eating and other disordered eating behaviors [i.e., diuretic/laxative use, excessive exercise, fasting, and vomiting (32)]. Binge eating was defined by DSM-5 as eating a large amount of food with loss of control in the past three months (45). Other questions related to disordered eating behaviors were framed around body weight or shape control. The percentage reporting binge eating was similar in both military/veterans and civilians, 71% and 76%, respectively. More military/veterans than civilians reported using diuretic/laxative (23 vs. 20%) and excessive exercise (45 vs. 40%) for weight management. Fasting and vomiting was reported by 42% and 22% of the military/veterans, respectively, similar to percentages reported in civilians. Unfortunately, military personnel and veterans were grouped together, and specific branches were undefined limiting applicability to active-duty service members. Additionally, a high percentage of respondents were female, who account for only ∼17% of all active-duty personnel (46).

In 575 soldiers (90% male), Allen et al. (2022) collected self-reported data using the Military Eating Behavior Survey (MEBS) on weight cycling and weight management practices. Weight cycling in MEBS was defined as having three or more weight fluctuations of ≥5% body weight, excluding pregnancy or illness with a dichotomous response of “yes” or “no” (33). One-third (33%) of soldiers reported participating in weight cycling, lower than observed in an analogous athlete group [combat sports, range = 40–90% (47)] but similar to general population (33). Although, since weight cycling definitions may vary by study, prevalence findings in this study may not be directly comparable. Allen et al. (2022) also asked participants to identify behaviors used to prepare for body composition assessment and compared responses from those who reported weight cycling (WC group) to those who did not (non-WC group). The four most prevalent practices reported were dieting (WC = 35 vs. non-WC = 21%), increased exercise (WC = 38 vs. non-WC = 26%), dehydration (WC = 18 vs. non-WC = 8%), and supplement use (WC = 18 vs. non-WC = 11%). A strength of this study was that it exclusively assessed active-duty personnel. However, soldiers only were included, and thus broader applicability to other branches should be done with caution.

### 4.3. Summary

The impact of body composition on health in military population has limited support demonstrating with differences between sexes noted, which can be contributed to female having higher %BF than males in general. Overall, higher adiposity was associated with inflammation (i.e., CRP and IL-19 = 8) and improved integrity of WM. VAT, however, was associated with decreased WM integrity, and thus, the type of fat (VAT vs. subcutaneous fat) may be more important. In previous reports, a higher BMI has been linked to poor cognitive health outcomes. A BMI classified as overweight/obese (>25 kg/m^2^) is also associated with increased MSKI risk, while research on the direct relationship between total body composition and injuries is lacking. Weight cycling and health, to our knowledge, have not been examined in military personnel. In two studies, weight cycling and disordered eating have been documented to occur in military populations as a means for weight control. Additional research is needed to exam the link among weight cycling, disordered eating, health, and weight control. The evaluation of existing body standards would benefit from this knowledge.

## 5. Conclusion and future directions

Although an argument has been made that body composition is an important determinant of military performance and health, the evidence-based research is lacking. For performance, LBM and/or LM/FM ratio may be a better indicator than %BF. A comprehensive study on weight cycling, disordered eating, and weight control would also be valuable to determine the prevalence of this issue and the impact on health and performance outcomes. This information along with performance data would support policy makers in determining if changes are warranted to existing body composition standards. To determine overall health, regular physical activity, total FFM, and VAT may be better metrics. Further research is needed, particularly in female service members and in other service branches (i.e., Air Force and Navy), to determine to the role of body composition as a whole (%BF, FM, and FFM) in military performance and health. Furthermore, the interaction between body composition and other characteristic such as race and ethnicity, postpartum status, and age should be examined.

## Author disclaimer

I am a military service member or employee of the U.S. Government. This work was prepared as part of my official duties. Title 17, U.S.C. §105 provides that copyright protection under this title is not available for any work of the U.S. Government. Title 17, U.S.C. §101 defines a U.S. Government work as work prepared by a military service member or employee of the U.S. Government as part of that person's official duties. The views expressed in this article are those of the authors and do not necessarily reflect the official policy or position of the Department of the Navy, Department of Defense, nor the U.S. Government.

## References

[1] Thomas DT Erdman KA Burke LM. American college of sports medicine joint position statement. Nutrition and athletic performance. Med Sci Sports Exerc. (2016) 48(3):543–68. 10.1249/mss.0000000000000852 26891166

[2] Peterson DD. History of the U.S. Navy body composition program. Mil Med. (2015) 180(1):91–6. 10.7205/milmed-d-14-00266 25562863

[3] Police SB Ruppert N. The US military’s battle with obesity. J Nutr Educ Behav. (2022) 54(5):475–80. 10.1016/j.jneb.2021.12.003 35534103

[4] *Weight Management: State of the Science and Opportunities for Military Programs*. Washington (DC)(2004). 25057674

[5] Ogden CL Carroll MD Curtin LR McDowell MA Tabak CJ Flegal KM. Prevalence of overweight and obesity in the United States, 1999–2004. JAMA. (2006) 295(13):1549–55. 10.1001/jama.295.13.1549 16595758

[6] National Health and Nutrition Examination Survey 2017–March 2020 Prepandemic Data Files Development of Files and Prevalence Estimates for Selected Health Outcomes, (2021). 10.15620/cdc:106273 PMC11513744 39380201

[7] Tompkins E. Obesity in the United States and effects on military recruiting. In: LOCW DC, editor. Washington, DC: Congressional Research Service (2022).

[8] Potter AW Tharion WJ Holden LD Pazmino A Looney DP Friedl KE. Circumference-based predictions of body fat revisited: preliminary results from a US marine corps body composition survey. Front Physiol. (2022) 13:1–10. 10.3389/fphys.2022.868627 PMC9008774 35432005

[9] Friedl K. Body composition: Health and performance in exercise and sports. In: Lukaski HC, editor. Boca Raton, FL: CRC Press. (2017). p. 285–306.

[10] Mountjoy M Sundgot-Borgen JK Burke LM Ackerman KE Blauwet C Constantini N Ioc consensus statement on relative energy deficiency in sport (red-S): 2018 update. Br J Sports Med. (2018) 52(11):687–97. 10.1136/bjsports-2018-099193 29773536

[11] Sundgot-Borgen J Meyer NL Lohman TG Ackland TR Maughan RJ Stewart AD How to minimise the health risks to athletes who compete in weight-sensitive sports review and position statement on behalf of the ad hoc research working group on body composition, health and performance, under the auspices of the ioc medical commission. Br J Sports Med. (2013) 47(16):1012–22. 10.1136/bjsports-2013-092966 24115480

[12] Abt JP Perlsweig K Nagai T Sell TC Wirt MD Lephart SM. Effects of age and military service on strength and physiological characteristics of U.S. Army soldiers. Mil Med. (2016) 181(2):173–9. 10.7205/milmed-d-15-00036 26837087

[13] Crawford K Fleishman K Abt JP Sell TC Lovalekar M Nagai T Less body fat improves physical and physiological performance in army soldiers. Mil Med. (2011) 176(1):35–43. 10.7205/milmed-d-10-00003 21305957

[14] Orantes-Gonzalez E Heredia-Jimenez J Escabias M. Body mass index and aerobic capacity: the key variables for good performance in soldiers. Eur J Sport Sci. (2022) 22(10):1467–74. 10.1080/17461391.2021.1956599 34259126

[15] Pihlainen K Santtila M Häkkinen K Kyröläinen H. Associations of physical fitness and body composition characteristics with simulated military task performance. J Strength Cond Res. (2018) 32(4):1089–98. 10.1519/jsc.0000000000001921 28549046

[16] Beck B Middleton KJ Billing DC Caldwell JN Carstairs GL. Understanding anthropometric characteristics associated with performance in manual lifting tasks. J Strength Cond Res. (2019) 33(3):755–61. 10.1519/jsc.0000000000002113 28682931

[17] Allison KF Keenan KA Lovalekar M Mi Q Beals K Coleman L Fight load index and body composition are most associated with combat fitness in female marines. J Sci Med Sport. (2019) 22(4):494–9. 10.1016/j.jsams.2018.10.014 30448087

[18] Royer SD Thomas DT Winters JD Abt JP Best S Poploski KM Physical, physiological, and dietary comparisons between marine corps forces special operations command critical skills operators and enablers. Mil Med. (2018) 183(11/12):e341–e7. 10.1093/milmed/usy049 29635381

[19] Sporis G Jukić I Bok D Vuleta D Jr. Harasin D. Impact of body composition on performance in fitness tests among personnel of the Croatian navy. Coll Antropol (2011) 35(2):335–9. PMID: . 21755699

[20] Ricciardi R Deuster PA Talbot LA. Effects of gender and body adiposity on physiological responses to physical work while wearing body armor. Mil Med. (2007) 172(7):743–8. 10.7205/milmed.172.7.743 17691688

[21] Holmes CJ Racette SB. The utility of body composition assessment in nutrition and clinical practice: an overview of current methodology. Nutrients. (2021) 13(8):2493. 10.3390/nu13082493 34444653 PMC8399582

[22] Lukaski HC. Body composition: Health and performance in exercise and sport. Boca Raton: CRC Press/Taylor & Francis Group (2017). xi, 388 pages p.

[23] Ansdell P Thomas K Hicks KM Hunter SK Howatson G Goodall S. Physiological sex differences affect the integrative response to exercise: acute and chronic implications. Exp Physiol. (2020) 105(12):2007–21. 10.1113/ep088548 33002256

[24] Jackson AS Pollock ML. Generalized equations for predicting body density of men. Br J Nutr. (1978) 40(3):497–504. 10.1079/bjn19780152 718832

[25] Potter AWN Lyndsey J Pazmino A Soto LD Hancock JW Looney DP US marine corps body composition and military appearance program (Bcmap) study. Natick, MA: Medicine USARIoE (2022).

[26] Tingelstad HC Filion LG Martin J Spivock M Tang V Haman F. Levels of circulating cortisol and cytokines in members of the Canadian Armed Forces: associations with age, sex, and anthropometry. Appl Physiol Nutr Metab. (2018) 43(5):445–52. 10.1139/apnm-2017-0551 29200312

[27] Cárdenas D Madinabeitia I Vera J Perales JC García-Ramos A Ortega E Strength, affect regulation, and subcortical morphology in military pilots. Med Sci Sports Exerc. (2018) 50(4):722–8. 10.1249/mss.0000000000001500 29166323

[28] Bertrandt J Szarska E Łakomy R Lepionka T Anyżewska A Lorenz K An attempt to utilize the body composition analyzer and the functional movement screen (Fms) test to determine injury risk in soldiers. Mil Med. (2020) 185(7-8):e1128–e33. 10.1093/milmed/usz476 32314793

[29] Hernández LM Coffin SD Taylor MK. Greater fitness is associated with improved functional movement characteristics in explosive ordnance disposal technicians. J Strength Cond Res. (2020) 36(6):1731–7. 10.1519/jsc.0000000000003704 32639372

[30] Heebner NR Abt JP Lovalekar M Beals K Sell TC Morgan J Physical and performance characteristics related to unintentional musculoskeletal injury in special forces operators: a prospective analysis. J Athl Train. (2017) 52(12):1153–60. 10.4085/1062-6050-52.12.22 29227730 PMC5759699

[31] Keenan KA Wohleber MF Perlsweig KA Baldwin TM Caviston M Lovalekar M Association of prospective lower extremity musculoskeletal injury and musculoskeletal, balance, and physiological characteristics in special operations forces. J Sci Med Sport. (2017) 20(Suppl 4):S34–s9. 10.1016/j.jsams.2017.09.002 28958636

[32] Flatt RE Norman E Thornton LM Fitzsimmons-Craft EE Balantekin KN Smolar L Eating disorder behaviors and treatment seeking in self-identified military personnel and veterans: results of the national eating disorders association online screening. Eat Behav. (2021) 43:101562. 10.1016/j.eatbeh.2021.101562 34534875 PMC8952181

[33] Allen JT Jayne J Karl JP McGraw SM O'Connor K DiChiara A Weight management behaviours mediate the relationship between weight cycling, bmi and diet quality among US army soldiers. Br J Nutr. (2022) 128(3):569–76. 10.1017/s000711452100338x 34470676

[34] Dos Santos Bunn P de Oliveira Meireles F de Souza Sodré R Rodrigues AI da Silva EB. Risk factors for musculoskeletal injuries in military personnel: a systematic review with meta-analysis. Int Arch Occup Environ Health. (2021) 94(6):1173–89. 10.1007/s00420-021-01700-3 33987772

[35] Hruby A Hill OT Bulathsinhala L McKinnon CJ Montain SJ Young AJ Trends in overweight and obesity in soldiers entering the US army, 1989-2012. Obesity (Silver Spring). (2015) 23(3):662–70. 10.1002/oby.20978 25611465

[36] Cowan DN Bedno SA Urban N Yi B Niebuhr DW. Musculoskeletal injuries among overweight army trainees: incidence and health care utilization. Occup Med (Lond). (2011) 61(4):247–52. 10.1093/occmed/kqr028 21482621

[37] Wearing SC Hennig EM Byrne NM Steele JR Hills AP. Musculoskeletal disorders associated with obesity: a biomechanical perspective. Obes Rev. (2006) 7(3):239–50. 10.1111/j.1467-789X.2006.00251.x 16866972

[38] Fothergill E Guo J Howard L Kerns JC Knuth ND Brychta R Persistent metabolic adaptation 6 years after “the biggest loser” competition. Obesity (Silver Spring). (2016) 24(8):1612–9. 10.1002/oby.21538 27136388 PMC4989512

[39] Rossi AP Rubele S Calugi S Caliari C Pedelini F Soave F Weight cycling as a risk factor for low muscle mass and strength in a population of males and females with obesity. Obesity (Silver Spring). (2019) 27(7):1068–75. 10.1002/oby.22493 31231958

[40] Zou H Yin P Liu L Liu W Zhang Z Yang Y Body-weight fluctuation was associated with increased risk for cardiovascular disease, all-cause and cardiovascular mortality: a systematic review and meta-analysis. Front Endocrinol (Lausanne). (2019) 10:728. 10.3389/fendo.2019.00728 31787929 PMC6856014

[41] Zou H Yin P Liu L Duan W Li P Yang Y Association between weight cycling and risk of developing diabetes in adults: a systematic review and meta-analysis. J Diabetes Investig. (2021) 12(4):625–32. 10.1111/jdi.13380 32745374 PMC8015818

[42] Cao V Makarem N Maguire M Samayoa I Xi H Liang C History of weight cycling is prospectively associated with shorter and poorer-quality sleep and higher sleep apnea risk in diverse US women. J Cardiovasc Nurs. (2021) 36(6):573–81. 10.1097/jcn.0000000000000818 33938536 PMC8601765

[43] Cialdella-Kam L Kulpins D Manore M. Vegetarian, gluten-free, and energy restricted diets in female athletes. Sports. (2016) 4(4):50. 10.3390/sports4040050 29910298 PMC5968895

[44] Nattiv A Loucks AB Manore MM Sanborn CF Sundgot-Borgen J Warren MP. American College of sports medicine position stand. The female athlete triad. Med Sci Sports Exercise. (2007) 39(10):1867–82. 10.1249/mss.0b013e318149f111 17909417

[45] American Psychiatric Association., American Psychiatric Association. DSM-5 Task force. Diagnostic and statistical manual of mental disorders: Dsm-5. 5th ed. Washington, D.C: American Psychiatric Association (2013). xliv, 947.

[46] 2021 *Demographics: Profile of the Military Community*. In: the Department of Defense (DoD) OotDASoDfMCaFPOMF, editor. Miitary OneSource.

[47] Miles-Chan JL Isacco L. Weight cycling practices in sport: a risk factor for later obesity? Obes Rev. (2021) 22(S2):e13188. 10.1111/obr.13188 33372395

